# Beyond Palliation: Therapeutic Applications of ^153^Samarium-EDTMP

**DOI:** 10.4172/2161-1459.1000131

**Published:** 2013-06

**Authors:** Breelyn A. Wilky, David M. Loeb

**Affiliations:** 1 Department of Oncology, Johns Hopkins University School of Medicine, Baltimore, Maryland; 2 Department of Pediatric Oncology, Johns Hopkins University School of Medicine, Baltimore, Maryland

**Keywords:** Samarium, Radiopharmaceutical, Bone Metastases, Osteosarcoma

## Abstract

Primary and metastatic malignant bone lesions result in significant pain and disability in oncology patients. Targeted bone-seeking radioisotopes including ^153^Samarium ethylene-diamine-tetramethylene-phosphonic acid (^153^Sm-EDTMP) have been shown to effectively palliate bone pain, often when external beam radiotherapy (EBRT) is not feasible. However, recent evidence also suggests ^153^Sm-EDTMP has cytotoxic activity either alone or in combination with chemotherapy or EBRT. ^153^Sm-EDTMP may be useful as anti-neoplastic therapy apart from pain palliation in a variety of malignancies. For prostate cancer patients, several phase I and II clinical trials have shown that combined ^153^Sm-EDTMP and docetaxel-based chemotherapy can result in >50% decrease in prostate-specific antigen with manageable myelosuppression. In hematologic malignancies, ^153^Sm-EDTMP produced clinical responses when combined with bortezomib in multiple myeloma. ^153^Sm-EDTMP also can be used with myeloablative chemotherapy for marrow conditioning prior to stem cell transplant. In osteosarcoma, ^153^Sm-EDTMP infusion delivers radiation to multiple unresectable lesions simultaneously and provides local cytotoxicity without soft tissue damage that can be combined with chemotherapy or radiation. Prior to routine incorporation of ^153^Sm-EDTMP into therapeutic regimens, we must learn how to ensure optimal delivery to tumors, determine which patients are likely to benefit, improve our ability to assess clinical response in bone lesions and further evaluate the efficacy ^153^Sm-EDTMP in combination with chemotherapy, radiation and novel targeted agents.

## Introduction

Malignant bone lesions are widely encountered in medical oncology patients and related pain and skeletal events, such as pathologic fracture, spinal cord compression, hypercalcemia, pancytopenia from bone marrow infiltration and immobility lead to poor performance status, impaired quality of life and inability to tolerate further treatment [1]. Three of the four most common cancers in the United States regularly metastasize to bone and multiple myeloma (MM) patients develop bone lesions up to 95% of the time [2]. While external beam radiotherapy (EBRT) can be effective for painful or structurally problematic lesions, local and systemic side effects including soft tissue injury, myelosuppression or fatigue can be intolerable. Additionally, even modern conformal techniques such as intensity-modulated radiotherapy (IMRT) have limited utility in the setting of multiple or diffuse lesions. Another challenge is local management of unresectable osteosarcoma, the most common primary bone tumor in children and young adults. Radiation therapy has historically not been useful, as the doses required for tumoricidal activity (60–80 Gy) often exceed local tissue tolerance, particularly in the axial skeleton [3–5]. Without effective local control, cure rates are dismal [6,7].

Targeted “bone-seeking” radioisotope delivery has been pursued as an alternative to EBRT for treatment of malignant bone lesions. Two are approved in Europe and the United States (^89^strontium and ^153^samarium) and others are still in the experimental phase but appear promising (^186^rhenium, ^188^rhenium, ^223^radium, ^166^holmium). Of the approved radiopharmaceuticals, ^153^samarium ethylene-diamine-tetramethylene-phosphonic acid (^153^Sm-EDTMP) is more clinically useful due to a shorter half-life. This remarkably well-tolerated radiopharmaceutical is easily administered and displays impressive specificity for bone lesions with toxicity limited to transient myelosuppression. In phase III clinical trials, 60–80% of patients with metastatic prostate or breast cancer have reported relief of pain within days of administration; the mechanism of pain relief is still unclear [8]. While ^153^Sm-EDTMP is approved by the Food and Drug Administration (FDA) to treat pain in osteoblastic lesions that enhance on ^99m^Technetium-MDP (^99m^Tc) bone scan, the use of ^153^Sm-EDTMP as a primary or adjunctive cytotoxic strategy is just beginning to be studied. In this review we will highlight the novel applications of ^153^Sm-EDTMP currently being explored in metastatic solid tumors, hematologic malignancies and osteosarcoma and discuss some of the obstacles to determining optimal use.

## Properties of ^153^Sm-EDTMP

The physical and chemical properties of ^153^Sm-EDTMP have been well described and recently reviewed [9–14]. In brief, ^153^Sm-EDTMP is created by neutron capture of ^152^Sm oxide and then chelated to the EDTMP moiety. ^153^Sm emits a low energy beta particle at maximum energies of 640, 710 and 810 keV with an average beta particle energy of 233 keV [9]. These particles penetrate tissue only over a relatively short distance of 1–2 mm, allowing the delivery of high doses of radiation to the tumor while sparing surrounding normal tissue. A medium energy photon (103 keV) is also given off by the ^153^Sm and allows for standard scintigraphic imaging to monitor delivery of radiation to the tumor. EDTMP (and its pentasodium salt, lexidronam) is structurally related to the chelating agent methylene diphosphonate (MDP) that is complexed to ^99m^Tc for conventional bone imaging. EDTMP binds to hydroxyapatite found in areas of new bone formation [13,15], with fivefold increased uptake in lesions compared to normal bone. A variable portion of the administered dose remains complexed to bone with minimal uptake in extraskeletal soft tissue [12]. Remaining activity is excreted through the kidneys, which is almost entirely completed within 8 hours of administration. The physical half-life is 46 hours, with radioactive decay of greater than 90% within one week [9].

A variety of doses and schedules have been reported in the literature. In general, palliative doses range from 0.5 to 2.5 mCi/kg (18.5–92.5 MBq/kg). With these doses, hematologic nadir occurs 3–5 weeks post-treatment and includes grade 0–2 leukopenia, anemia and thrombocytopenia. Neutropenia can easily be managed using growth factors, with neutropenic fever and sepsis quite rare. Hematologic recovery is generally complete by 8 weeks. More prolonged and pronounced myelosuppression can be seen in heavily pretreated patients or patients with other bone marrow comorbidities [16], but with appropriate monitoring patients have safely received ^153^Sm-EDTMP before or after chemotherapy and EBRT [17,18]. A dose of 3 mCi/kg (111 MBq/kg) causes frequent grade III–IV thrombocytopenia and neutropenia [19,20]. Repeated schedules of ^153^Sm-EDTMP have been described in several studies without cumulative myelosuppression [19,21–24]. For example, patients with metastatic castrate-resistant prostate cancer (CRPC) tolerated three cycles of 2 mCi/kg (74 MBq/ kg) every 16 weeks with 7 episodes of reversible grade III neutropenia out of 18 patients [21]. Doses as high as 30 mCi/kg (1110 MBq/kg) have been administered, followed by autologous stem cell transplant (SCT) infused 14 days after ^153^Sm-EDTMP treatment, once residual total body radioactivity has diminished to <3.6 mCi/kg (133.2 MBq/kg). No systemic toxicity apart from expected grade IV myelosuppression has been observed [25–29]. ^153^Sm-EDTMP has been shown to be highly concentrated and toxic to physeal growth plates *in vitro* [30] and is not approved for use in children, although in poor prognosis scenarios such as palliation of end-stage metastases or local management of an unresectable osteosarcoma, this concern may be less relevant.

In evaluating clinical efficacy of ^153^Sm-EDTMP, it is critical to accurately measure absorbed radioactivity delivered to the target lesion. Traditionally, a scintigraphic camera focused on the 103 keV photopeak obtains whole body planar images and by applying MIRD formalism, the tumor absorbed dose in Gy is calculated [31–33]. When post-^153^Sm-EDTMP dosimetry is performed, the absorbed dose at tumors can be highly variable between patients and even among tumors within the same patient. Early pharmacokinetic studies showed ^153^Sm-EDTMP uptake at the bone surfaces ranged from 40–95% of the administered activity [34]. In a recent study by Vigna, absorbed activity in prostate and breast metastatic lesions following 1 mCi/kg (37 MBq/ kg) was 2.1 Gy (range: 0.7–3.5) at the red marrow and 11.5 Gy (range: 5.0–18.4) at the bone surface [35]. In seven patients with osteosarcoma receiving high-dose ^153^Sm-EDTMP Anderson reported absorbed doses from 39 to 241 Gy, median 189 Gy, following 30 mCi/kg (1110 MBq/ kg) [25]. However another study of sixteen osteosarcoma tumors showed far lower absorbed doses ranging from 1.8 to 66.2 Gy, median of 25.2 Gy, following 6 mCi/kg (222 MBq/kg) [36].

Possible explanations for this variability include differences in the proportion of osteoblastic to osteolytic activity within a lesion, bone density, tumor blood flow, intratumoral necrosis or hypoxia and the number or size of metastatic lesions [9,37,38]. Different histologies may be more amenable to uptake; absorbed dose in bone and red marrow was significantly higher in prostate cancer than in breast cancer patients [35]. Additionally, in tumors with heterogeneous uptake, two-dimensional dosimetry often overestimates delivered radiation based on superimposition of signal [39,40]. More modern techniques utilize three-dimensional data acquired using SPECT/ CT, enabling far more precise imaging of heterogeneous radiation absorption within the tumor and correction of spillout effect [41]. Regardless of dosimetry technique, a solution to control for variable uptake is the use of tandem dosing of ^153^Sm-EDTMP. An initial tracer dose is given to measure uptake, followed by a subsequent higher dose; this method is regularly used in myeloablative regimens prior to transplant [28,29,34,36]. Importantly, most early studies investigating ^153^Sm-EDTMP do not report dosimetric data for patients. In many pain palliation trials, improvement in pain scores and a remarkably consistent degree of hematologic toxicity is observed across the range of palliative dosing without a consistent dose-response relationship [19]. This inconsistency could certainly be explained, at least in part, by variable uptake.

Despite this variability, ^153^Sm-EDTMP clearly has specific analgesic activity for bone pain and is licensed by the FDA for treatment of pain resulting from skeletal metastases that enhance on ^99m^Tc bone scan. Several randomized, placebo-controlled clinical trials showed significantly decreased pain scores after the use of 0.5 or 1 mCi/ kg (18.5–37 MBQ/kg) compared to placebo [42–46]. The majority of patients studied have been men with CRPC, but women with breast cancer were also shown to benefit [44,47–50]. Multiple reviews have recently revisited these and other trials [51,52]. In the Cochrane review update, potential bias was described in many trials measuring subjective pain reporting and no measurable decrease in opiate requirements was demonstrated; however, the general consensus remains that ^153^Sm-EDTMP can provide significant pain relief when more traditional means fail (such as EBRT), but at the expense of hematologic toxicity [51]. Around 60–80% of CRPC and breast cancer patients report improved pain control, often for several months, as well as improved performance status and quality of life scores, particularly for prostate cancer patients [52,53].

For cancers other than prostate and breast, data for pain palliation with ^153^Sm-EDTMP is quite limited. Studies of prostate and breast cancer patients occasionally include very small numbers of patients with bladder, GI, thyroid and parathyroid, ovarian, germ cell, head and neck and unknown primary tumors, but pain or clinical response outcomes are rarely reported for these specific subtypes [12,19,44,46,54–59]. By extrapolating anecdotal comments from these studies, patients with lung cancer treated with ^153^Sm-EDTMP seemed to be less likely to experience pain relief compared to prostate or breast cancers. Reports of excellent pain relief occurred in neuroendocrine-type tumors including small cell lung cancer and carcinoid tumors [12].

Even within larger studies of prostate and breast cancer patients, reports of clinical response outcomes such as radiographic changes, improvement in survival, or decreased biomarkers (including prostate-specific antigen (PSA), cancer antigen 15–3 (CA 15–3), or alkaline phosphatase (AP)) are quite sparse. When present, data are often uninterpretable due to lack of dosimetry data to confirm equal exposure of tumors to cytotoxic radiation. Additionally, assessment of radiographic changes in bone tumors can be challenging and serum biomarkers have not been demonstrated to act as a surrogate for survival, particularly in patients who also have non-osseous disease. Essentially no information is available for these outcomes in non-prostate, non-breast solid tumor histologies. Therefore, much remains to be learned about the therapeutic potential of ^153^Sm-EDTMP. In the remainder of this review, we will focus on the emerging evidence for cytotoxic activity of ^153^Sm-EDTMP therapy, either alone or in combination with chemotherapy or EBRT and evaluate outcomes apart from pain palliation in three areas: metastatic solid tumors, hematologic malignancies and primary bone tumors.

## ^153^Sm-EDTMP for metastatic solid tumors

Despite the limitations of early studies evaluating ^153^Sm-EDTMP, evidence exists for a small but critical proportion of patients who not only experience improvement in pain, but also in disease burden. Since many patients with metastatic solid tumors are end-stage, or go on to receive other systemic therapy after ^153^Sm-EDTMP, few studies evaluate overall or even progression-free survival. However, some report post-treatment bone scans and serum biomarkers. In Turner’s initial study of 35 patients (15 prostate, 10 breast, 10 other) who received dosimetry-confirmed exposure to 100–280 cGy with ^153^Sm-EDTMP, 15 of 34 evaluable patient showed improvement or stabilization in bone scans three months after therapy [34]. No changes were seen in AP levels or PSA (where applicable) and no dose-response effect could be determined due to small sample size. In a follow-up study of 23 patients with prostate, breast and other tumors who received ^153^Sm-EDTMP dosing to 2 Gy, Turner reported that patients who were retreated after hematologic recovery showed improvement in overall survival relative to patients who received only one dose (9 months vs. 4 months) [24]. They note that “improvements in bone scans were seen, but this was not a consistent finding,” and no correlation of bone scan results with survival was provided. Since healthier patients may have been more likely to receive a second infusion, the survival benefit could be biased, however with “unchanged” bone scans, some patients could have derived clinical benefit from stabilization of disease. Two other studies reported similar findings but did not include dosimetry or survival data – despite improvement in pain control, patients showed “no changes” in bone scans, PSA, or AP levels [57,60].

In prostate cancer-specific studies, results may be a bit more encouraging. In a study of 32 men with CRPC who received 1.1 mCi/ kg (40 MBq/kg) ^153^Sm-EDTMP, 88% of patients had an improved or stable bone scan one month post treatment and 78% of patients had improved or stable scans at 3 months [61]. There was no significant change in AP or prostatic acid phosphatase (PAP) and PSA was significantly increased at 3 months. Another study of 82 patients (75% prostate) treated in three separate ^153^Sm-EDTMP dose cohorts (0.75, 1.5 and 3 mCi/kg [27.75, 55.5, 111 MBq/kg]) found only three patients to have any degree of regression of metastases on bone scan, but a median decrease in PSA of 21–28% regardless of dose [19]. Lastly, 52 patients with CRPC received ^153^Sm-EDTMP in doses from 0.5–3 mCi/ kg (18.5–111 MBq/kg) in a phase I/II dose escalation study, with 40 of these patients receiving 1.0 mCi/kg (37 MBq/kg) or 2.5 mCi/kg (92.5 MBq/kg) as phase II cohorts [20]. One month following treatment, 17 of all 52 patients showed >25% decrease in PSA, 32 of 52 showed >25% decrease in PAP and 36 of 50 evaluable men had stable or improved bone scans. Within the phase II cohorts, more patients showed a PSA decrease >25% at 1 and 2 months post-therapy in the 2.5 mCi/kg group compared to the 1.0 mCi/kg group (1 month: 50% vs 10%, 2 month: 42% vs 7%). A median survival benefit of 3 months was seen in patients treated with 2.5 mCi/kg compared to the 1.0 mCi/kg cohort, reportedly statistically significant though no p-value was provided. While these studies in prostate cancer suggest clinical benefit apart from pain palliation, none of them included dosimetry to permit controlled comparisons of actual absorbed dose.

Very promising data have recently emerged from studies combining ^153^Sm-EDTMP with docetaxel-based chemotherapy in CRPC. Four phase I studies have evaluated docetaxel 25 mg/m^2^ weekly or up to 75 mg/m^2^ every three weeks with one or two ^153^Sm-EDTMP 0.5–1 mCi/kg (18.5–37 MBq/kg) infusions, based on hematologic recovery (as frequently as every 4 weeks) [62–65]. Hematologic toxicity was surprisingly manageable; most studies included concurrent steroid use and growth factor support. Grade III/IV anemia, leukopenia, or thrombocytopenia occurred occasionally but were reversible, with the exception of one patient who died from neutropenic fever and sepsis [65]. Three of the four studies reported at least 50% of patients achieving >50% PSA reduction, even in some taxane-resistant patients. One study reported stable or improved bone scans in 7 of 9 patients [65] and another showed that 1 of 6 patients with measurable disease achieved a partial response by RECIST criteria [63]. A phase II study treated 43 CRPC patients with an induction regimen of docetaxel and estramustine and those with an initial PSA response (n=41) received consolidation therapy with weekly docetaxel 20 mg/m^2^ and ^153^Sm-EDTMP 1 mCi/kg (37 MBq/kg) [66]. 34 patients received 5 of 6 docetaxel infusions during the consolidation period. 77% of patients had a PSA response overall and the median percent of patients with >30% decline was 81%. PSA-based progression-free survival (PFS) was 6.4 months, clinical PFS was 15 months and median overall survival was 29 months. No grade IV myelosuppression occurred and patients who failed therapy were able to receive subsequent chemotherapy suggesting that the regimen did not result in prohibitive marrow toxicity.

In non-metastatic prostate cancer, 29 patients with localized, high-risk disease (PSA >20, Gleason score>8, or >T3 lesion) received one month of androgen deprivation therapy (ADT), followed by ^153^Sm-EDTMP (escalating dose from 0.25 to 2 mCi/kg [9.25–74 MBq/kg]) [67]. Twelve weeks after ^153^Sm-EDTMP, patients received EBRT including 46.8 Gy to pelvic lymphatics with prostatic boost to 70.2 Gy. Grade III hematologic toxicity occurred in 2 patients and one grade III dermatitis was noted. After median follow-up of 23 months, 12 of 18 patients had a PSA <0.2. Three of 18 went back on ADT for sharply rising PSA. In the adjuvant setting, a phase II study is ongoing for patients with high-risk, non-metastatic prostate cancer with a rising PSA following radical prostatectomy. Patients in this trial will receive ^153^Sm-EDTMP 2 mCi/kg (74 MBq/kg), followed 12 weeks later by IMRT 70.2 Gy to the prostatic fossa (NCT 013170043, NCT 00551525) [68].

In summary, several studies suggest anti-tumor activity for ^153^Sm-EDTMP in prostate cancer as a single-agent and phase I studies have shown that docetaxel-based chemotherapy can also be given with ^153^Sm-EDTMP with manageable myelosuppression. Further phase II evaluation is required for better understanding of how much additional benefit ^153^Sm-EDTMP provides apart from docetaxel and whether improved bone scans and PSA-defined progression-free survival correlate with overall survival benefit, particularly in patients with visceral disease as well. Larger numbers of patients will also help better define the incidence of grade III/IV myelotoxicity and complications such as neutropenic sepsis. Additionally, none of these trials included dosimetry to confirm uniform uptake of ^153^Sm-EDTMP to the tumors which will also help to clarify potential benefit. An interesting ongoing investigation in metastatic disease is a randomized phase 2.5 study for men with docetaxel-refractory CRPC wherein arm A receives ^153^Sm-EDTMP every 12 weeks and arm B receives ^153^Sm-EDTMP plus PSA/ Tricom vaccine therapy (NCT00450619) [68]. In-vitro observations have shown that exposure to ^153^Sm-EDTMP radiation may increase T-cell mediated killing by upregulation of surface molecules Fas, CEA, mucin-1, MHC Class I and ICAM-1. Additionally, LNCaP cells (prostate cancer cell line) were more susceptible to killing by cytotoxic lymphocytes specific for PSA, carcinoembryonic antigen and mucin-1 [69]. Further exploration of the immunologic aspects of ^153^Sm-EDTMP may suggest other tumors in which combined immunotherapy might be effective.

While incorporation of ^153^Sm-EDTMP as a cytotoxic component of prostate cancer therapy appears promising, very few studies have investigated similar strategies in other metastatic solid tumors. In a recent study of 43 breast cancer patients with osteoblastic or mixed osteoblastic/osteolytic bone lesions who received 1–1.5 mCi/kg (37–55 MBq/kg) ^153^Sm-EDTMP, bone scans three months later showed improvement in 12% and stable disease in 70% of patients [49]. Serum markers one month after treatment showed only a minimal decrease in AP which was not persistent at three months and bone-specific AP and CA 15-3 levels increased. A study from China reports that out of 76 metastatic breast cancer patients who received two doses of ^153^Sm-EDTMP, only 6 had regression of metastatic lesions and 16 had stable disease [50]. Apart from these two studies, neither of which report dosimetry or survival, data regarding clinical outcomes for breast cancer patients treated with ^153^Sm-EDTMP are minimal. No studies have combined ^153^Sm-EDTMP with chemotherapy or radiation for breast cancer patients. Fortunately, a trial for metastatic breast cancer patients of high-dose ^153^Sm-EDTMP followed by autologous SCT is currently ongoing. The primary outcome to be evaluated is progression-free survival and secondary outcomes include pain relief, overall survival and safety (NCT 00429507) [68]. Outcomes for other tumor histologies such as lung cancer can only be extrapolated from larger trials and any reported effect is probably best considered anecdotal.

## ^153^Sm-EDTMP for hematologic malignancies

Reported use of ^153^Sm-EDTMP in hematologic malignancies falls into two paradigms - low-dose as a single agent for pain control or in combination with radiosensitizing drugs, or high-dose with a myeloablative agent for pre-transplant conditioning. Thus far, specific anti-tumor activity outside of transplant has only been suggested in multiple myeloma. In vitro studies showed that myeloma cell lines treated with ^153^Sm-EDTMP showed a 50% decrease in clonogenic activity and mice who received treatment had improved median survival from 18 to 25 days (p<0.001) [70]. Despite the classical teaching that myeloma bone lesions are primarily osteolytic, some patients have enhancing lesions on bone scan that possess osteoblastic components, enabling ^153^Sm-EDTMP uptake. ^153^Sm-EDTMP improved pain in ^99m^Tc-avid myeloma bone lesions, as evidenced by a study of 10 patients with refractory disease who received ^153^Sm-EDTMP (total dose 54 mCi [2000 MBq] per infusion) every twelve weeks for 2 or 3 cycles, combined with monthly zoledronic acid [71]. Not only did pain levels decrease, but M-protein levels decreased in 4 of the 10 patients. Although the relative contribution to pain palliation from the bisphosphonate cannot be determined in this study, further data (discussed below) suggests that ^153^Sm-EDTMP may be superior to bisphosphonates for pain control.

Preclinical evidence supports that bortezomib acts as a radiosensitizer in myeloma, possibly through inhibitory effects on the NF-κB pathway which upregulates anti-apoptotic signaling following exposure to ionizing radiation [72]. Impressive synergistic killing was also seen when bortezomib was combined with ^153^Sm-EDTMP [70]. Based on these results, 24 heavily-pretreated myeloma patients, including 13 (54%) who had received prior bortezomib, were treated with escalating doses of ^153^Sm-EDTMP (up to 1 mCi/kg [37 MBq/kg]) and bortezomib (1.0 or 1.3 mg/m^2^) [73]. Bortezomib was administered on days 1, 4, 8 and 11 and ^153^Sm-EDTMP was given on day 3 every eight weeks. Seven patients received at least three cycles and five completed all planned four cycles, with 14 patients withdrawn from study for progression after the first cycle. MTD was 0.5 mCi/kg ^153^Sm-EDTMP with 1.3 mg/m^2^ bortezomib. Interesting, bortezomib dosed at 1.0 mg/ m^2^ with ^153^Sm-EDTMP 1.0 mCi/kg was well tolerated. Three complete responses and two minimal responses were seen; complete responders received higher doses of ^153^Sm-EDTMP but none had received prior bortezomib therapy, while minor responders received lower ^153^Sm-EDTMP doses and both had received prior bortezomib. No systemic toxicity was seen apart from grade III/IV myelosuppression (neutropenia 12%, TCP 8%). Dosimetry results were not included in either low-dose ^153^Sm-EDTMP study.

Multiple myeloma has also been treated with high-dose ^153^Sm-EDTMP in the transplant setting with mixed results [27–29,74,75]. In a phase II trial of 46 patients with newly-diagnosed and relapsed myeloma who received 40 Gy to bone marrow by ^153^Sm-EDTMP (confirmed with dosimetry), followed by infusion of melphalan 200 mg/m^2^ and autologous SCT, 15 patients achieved a complete remission, 12 achieved a very good partial remission and 18 achieved partial remission, with toxicity limited to expected myelosuppression [27]. When compared to a cohort of patients undergoing autologous SCT with melphalan alone, with median follow-up of 7.1 years, overall survival favored the ^153^Sm-EDTMP group, but no significant difference was seen in overall survival, progression-free survival or response rate. In contrast, 9 patients with myeloma received 35 Gy by ^153^Sm-EDTMP (confirmed with dosimetry), as well as 10 Gy EBRT to entire extremities (based on prior observations of poor appendicular uptake with ^153^Sm-EDTMP), followed by cyclophosphamide 50 mg/kg/day for four days in preparation for matched related donor SCT [75]. Only two patients surviving > 3 months achieved complete remission, five patients achieved partial remission and no response was seen in one. Transplant-related mortality was 11% at three months and median overall survival was 24 months. When this cohort was compared to control patients receiving allogeneic SCT with total body irradiation (TBI)/cyclophosphamide preparation, no difference was seen in median overall survival, but the ^153^Sm-EDTMP group had inferior response rate by univariate analysis (25 vs 74% P=0.032) and inferior progression-free survival (median PFS 12 vs 66 months, p=0.004). A third study evaluated ten patients with multiple hematologic malignancies, including seven patients with myeloma who received 35 Gy from ^153^Sm-EDTMP (confirmed by dosimetry), followed by melphalan 200 mg/m^2^ for autologous SCT (n=5) or cyclophosphamide 50 mg/m^2^/day for four days for matched related donor SCT (n=2) [29]. Pancytopenia occurred earlier and was more prolonged than that seen in traditional conditioning, but no dose-limiting hematologic toxicity occurred, even in one patient who had received a prior allogeneic transplant. Four of the seven patients with myeloma achieved complete remission and two achieved partial remission. The impact of these studies on current standard of care is unclear, as the role of SCT in myeloma is highly debated now in light of superior response rates that are achievable with modern therapies including bortezomib and lenalidomide; however, these studies do suggest that ^153^Sm-EDTMP can safely be used in combination with melphalan or cyclophosphamide as part of a transplant conditioning regimen.

^153^Sm-EDTMP has also been used successfully in the transplant setting for other hematologic malignancies. In Macfarlane’s study, the three non-myeloma patients included one with refractory anemia with excess blasts in transformation (RAEB-T), one with acute myeloid leukemia (AML) and one with refractory large granular lymphocyte leukemia (LGL) [29]. All three underwent allogeneic SCT with high-dose ^153^Sm-EDTMP followed by cyclophosphamide 50 mg/m^2^/day for 4 days. The patient with LGL achieved 100% donor engraftment and had no evidence of disease 100 days post-transplant. The patient with AML had an uneventful transplant course with 100% donor engraftment but died of recurrent disease 150 days after transplant. The patient with RAEB-T developed transplant-related complications (venoocclusive disease, pulmonary infiltrates, secondary graft failure and fungal infection) and remained platelet-dependent until death, through neutrophil recovery occurred rapidly. Given lack of ^153^Sm-EDTMP uptake in the liver, it was felt that the complications were not related to ^153^Sm-EDTMP. Another study reported the use of ^153^Sm-EDTMP in four pediatric patients with high-risk AML and contraindications to usual TBI conditioning [76]. In a similar fashion, they received ^153^Sm-EDTMP followed by melphalan or busulfan/cyclophosphamide for allogeneic SCT (n=3) or autologous SCT (n=1). Toxicity was similar to conditioning regimens that include TBI and all four patients showed pathologic and cytogenetic response following recovery, although only two of the four patients remained in a durable remission, with the other two dying from relapsed disease. Since AML-related malignancies carry a poor prognosis in the transplant setting, particularly in adult patients with underlying myelodysplasia, it remains unclear whether ^153^Sm-EDTMP impacts transplant outcomes in these patients, but it appears to be a safe and effective agent for myeloablation.

One final interesting use of ^153^Sm-EDTMP was for a young patient with severe POEMS syndrome, a plasma cell dyscrasia that results in polyneuropathy, organomegaly, endocrinopathies, monoclonal gammopathy and skin changes [77]. He received 3 mCi/kg (111 MBq/kg) ^153^Sm-EDTMP, followed by melphalan 100 mg/m^2^ for 2 days starting on day 20, followed by autologous SCT. Toxicities were similar to other autologous conditioning regimens and the patient had recovery of counts approximately 14 days following melphalan. Marked clinical improvement and lack of serum or urine M-protein was reported 23 months after transplant.

Based on the promising results in these studies, ^153^Sm-EDTMP has several potential applications in the treatment of hematologic malignancies: single-agent activity in myeloma for management of pain and possible M-protein response, combinatorial use in myeloma with bortezomib or other radiosensitizing chemotherapy and high-dose ^153^Sm-EDTMP as a component of pre-transplant conditioning regimens for a variety of malignancies. Given the favorable toxicity profile without the systemic toxicity of other chemotherapy, further exploration of these approaches is clearly warranted.

## ^153^Sm-EDTMP in osteosarcoma

Osteosarcoma is the most common primary bone tumor in children and young adults and localized disease can be cured with multiagent chemotherapy and aggressive surgical resection up to 75% of the time. When lesions are unresectable, patients invariably die from the disease. Radiation therapy has historically been of limited utility for local control due to the high doses (60–80 Gy) required for tumoricidal activity, which often exceeds local tissue tolerance especially in the axial skeleton. However, ^153^Sm-EDTMP is taken up avidly by most osteosarcomas and offers a means of delivering radiation to multiple lesions throughout the skeleton, without soft tissue toxicity.

The efficacy of ^153^Sm-EDTMP has been demonstrated in both murine and canine models of osteosarcoma. Winderen et al reported that ^153^Sm-EDTMP could effectively treat orthotopic human osteosarcoma implanted in immunodeficient mice [78]. In early studies of canine osteosarcoma, forty dogs with spontaneous osteosarcomas were treated with one or two doses of 1 mCi/kg (37 MBq/kg) ^153^Sm-EDTMP and seven had durable remissions [79]. Small lesions, metastatic lesions and lesions of the axial skeleton responded well, while large lesions with minimal tumor bone formation responded poorly. In another study, there was a complete remission in 1 of 9 dogs treated with 1 mCi/kg (37 MBq/kg) [80]. A dosimetry study in this animal model system showed that approximately 20 Gy were delivered to bone tumors by administration of 1–1.5 mCi/kg (37–55.5 MBq/kg) ^153^Sm-EDTMP [81]. A more recent study showed improvement of lameness in 63% of 35 dogs receiving between 1 and 4 doses of 1 mCi/kg (37 MBq/kg) ^153^Sm-EDTMP for primary bone tumors who were not candidates for other therapy [82].

The first report of a human patient treated for osteosarcoma with ^153^Sm-EDTMP was published in 1996 [83]. A 35-year-old man with a primary osteosarcoma of the first lumbar vertebra had a local relapse with significant pain and neurologic dysfunction related to spinal cord compression. He was treated with two doses of ^153^Sm-EDTMP, eight weeks apart, at approximately 1 mCi/kg (37 MBq/kg) per dose. He had significant improvement in neurologic function and resolution of his pain that lasted for six months. Additionally, a group from Munich reported treating six patients with unresectable localized or metastatic osteosarcoma with a combination of multi-agent chemotherapy, high-dose ^153^Sm-EDTMP followed by autologous SCT and EBRT [84]. Three patients who received all three modalities responded, including one patient who survived more than three years. More recently Anderson et al demonstrated that high dose ^153^Sm-EDTMP at 30 mCi/kg (1110 MBq/kg), followed by SCT for myelosuppression could deliver doses as high as 240 Gy to metastatic osteosarcoma lesions [25]. Thirty patients were treated and all of them had either a reduction in opiate requirement or complete resolution of their pain with no non-hematologic toxicity.

^153^Sm-EDTMP can also be combined with radiosensitizing chemotherapy in osteosarcoma. Fourteen heavily pretreated patients (between two to four prior chemotherapy regimens) with ^99m^Tc-avid osteosarcoma lesions received 30 mCi/kg ^153^Sm-EDTMP followed by gemcitabine [85]. Gemcitabine was initially dosed 250 mg/m^2^ daily for five doses starting on day 2, but when a patient developed severe mucositis, this was changed to a single dose of gemcitabine 1500 mg/m^2^ on day 2. SCT was infused on day 14 to correct grade IV hematologic toxicity. Of note, dosimetry was not reported in this study. After 6–8 weeks, there were six partial remissions, two mixed responses and six patients with progression. Serum AP decreased in six of eight patients and indicator lesions improved on imaging in 8 of 14 patients. In the 12 patients with follow-up of >1 year, the longest duration of response was 11 months. 11 of 14 patients relapsed locally and 3 of 14 developed new distant pulmonary metastases. In clinical practice, other radiosensitizing chemotherapies that are reported include high-dose methotrexate with leucovorin rescue, followed by ^153^Sm-EDTMP and gemcitabine [10]. Additionally, EBRT combined with chemotherapy and ^153^Sm-EDTMP has also been used with some success in a palliative setting [84,86–88].

Our group has completed a two-phase study of heavily pre-treated osteosarcoma patients who received tandem dosing of tracer and therapeutic infusions of ^153^Sm-EDTMP [26,36]. For the tracer infusion, patients initially received 1.0 – 1.4 mCi/kg (37–51.8 MBq/kg) ^153^Sm-EDTMP in the dose-finding portion of the study, or 1.2 mCi/kg (44.4 MBq/kg) thereafter, the MTD defined as hematologic recovery in six weeks. After dosimetry, patients received a treatment infusion of 6 mCi/kg (222 MBq/kg) followed by autologous SCT fourteen days later. Clinical response was determined by radiographic imaging with CT or MRI scanning. Six of the eleven patients treated with tandem doses showed transient radiographic stabilization, though all eventually progressed. The median time to progression was 79 days for the entire cohort and 142 days for the four patients who experienced disease stabilization after the higher dose. Two patients achieved prolonged survival (990 and 1432 days). As expected, toxicity was limited to myelosuppression.

We saw highly variable tumor absorbed doses (ranging from 1.8 to 66.2 Gy, with a median of 25.2 Gy after 6 mCi/kg [222 MBq/kg] ^153^Sm-EDTMP) but observed a linear correlation between tumor absorbed dose after the tracer and treatment doses. This suggests that based on dosimetry after a tracer dose, one can calculate the expected absorbed dose after treatment infusion. Several patients in the study underwent dosimetry using three-dimensional SPECT/CT analysis which showed that within the tumor, radiation uptake was quite heterogeneous. This suggests that in two-dimensional dosimetry, uptake in these tumors may be overestimated due to superimposed signal and spillover effect [41]. As we have described, three-dimensional SPECT/CT dosimetry data can be translated to effective biologic dose, converted to traditional EBRT fraction equivalents and incorporated into a subsequent IMRT treatment plan [89]. In this manner, after administration of maximal ^153^Sm-EDTMP with SCT, we could customize an IMRT treatment plan to combine with radiopharmaceutical exposure to reach summative tumoricidal dosing (60–80 Gy) and minimize soft tissue toxicity from the EBRT component.

In summary, ^153^Sm-EDTMP appears to have cytotoxic activity in osteosarcoma and is safe and tolerable as a single agent as well as in combination with chemotherapy and radiation. The ongoing challenge is ensuring adequate delivery and overcoming the innate variability of tumor uptake.

## ^153^Sm-EDTMP and bisphosphonates

A final consideration in treatment of malignant bone lesions is how ^153^Sm-EDTMP fits in with bisphosphonate therapy, a cornerstone in the prevention of skeletal-related events and improvement of bone pain. In one study comparing the two agents, 18 patients with bone metastases from breast, prostate, lung and GI primaries were treated with either ^153^Sm-EDTMP 1 mCi/kg (37 MBq/kg) or pamidronate 30 mg IV. After four months, 77.8% of patients receiving ^153^Sm-EDTMP reported an effective pain response, compared to 44.4% of the pamidronate group; no statistical analysis was performed on the two groups [58]. There are several reports of combined bisphosphonate therapy and ^153^Sm-EDTMP without increased toxicity [71,90]. For example, a patient with metastatic prostate cancer received monthly zoledronic acid and two infusions of ^153^Sm-EDTMP over a six-month period. He experienced marked improvement in pain and his bone scan, PSA and bone-specific AP levels all improved [90]. Hematologic toxicity was mild and no significant renal toxicity or hypercalcemia was seen. There is a theoretical concern that bisphosphonates might compete with ^153^Sm-EDTMP in binding to bone, interfering with ^153^Sm-EDTMP uptake. A phase I study evaluated urinary excretion of ^153^Sm-EDTMP in patients receiving combined bisphosphonates and repeated palliative-dose ^153^Sm-EDTMP and found that bisphosphonate therapy did not lead to increased excretion of ^153^Sm-EDTMP [91]. Another group measured skeletal uptake of ^153^Sm-EDTMP using scintigraphy and noted no difference in uptake before and after treatment with pamidronate [92]. Thus, it appears that patients with bone metastases may receive both bisphosphonates and ^153^Sm-EDTMP if appropriate without concern for increased toxicity, or impaired skeletal uptake of either agent.

Although bisphosphonates have been proven effective in the prevention of skeletal related events in patients with known bone metastases, they are not proven to be effective in the prevention of bone disease and preclinical suggestion of direct cytotoxicity has not been shown in clinical trials. Whether the combination of ^153^Sm-EDTMP and bisphosphonates might have synergistic therapeutic benefit requires further investigation. There are two ongoing studies that will help to clarify the role of combined therapy. One study is evaluating safety, tolerability and efficacy in relief of bone pain in multiple myeloma patients receiving ^153^Sm-EDTMP plus bisphosphonate therapy (NCT00482378) [68]. The second is a randomized phase III trial comparing zoledronic acid with calcium and vitamin D to either ^89^Strontium or ^153^Sm-EDTMP in combination with zoledronic acid, calcium and vitamin D in patients with bone metastases from prostate, breast, or lung primaries (NCT00365105) [68]. At this time, both therapies appear to be effective options for palliation of pain.

## Conclusion

Effective management of malignant bone lesions is an important clinical problem in order to prevent fractures and other skeletal-related events, avoid progressive electrolyte abnormalities and most importantly, alleviate pain that limits quality of life and functional status for patients. While patients with metastatic solid tumors more often die from diffuse visceral metastasis, a high burden of bone disease often precedes this and is an important opportunity for delay of progression and improvement of quality of life. In primary bone tumors such as osteosarcoma, the ability to provide definitive local therapy is limited for patients with multiple lesions, unresectable metastases, or tumors in locations that prohibit tumoricidal-dose EBRT. Additionally, bone metastases independently portend a poor prognosis in many other tumors of children and young adults including Ewing’s sarcoma, rhabdomyosarcoma and neuroblastoma. ^153^Sm-EDTMP is highly effective in palliating pain resulting from osteoblastic lesions. Moreover, despite the limitations of study design, ^153^Sm-EDTMP appears to have potential cytotoxic effect for some bone tumors, either as a single-agent or in combination with chemotherapy or radiation. In light of the favorable toxicity profile and ease of administration by nuclear medicine departments, a trial of ^153^Sm-EDTMP therapy is a worthy option for patients with painful malignant bone lesions. The real challenge is learning how to optimize the therapeutic potential of ^153^Sm-EDTMP apart from pain palliation, as many questions remain unanswered.

The first question is how to objectively determine clinical benefit in patients with malignant bone lesions. Subjective pain ratings, quality of life scores and functional status measures remain the most clinically relevant outcomes, but they are prone to bias and difficult to objectively evaluate. Radiographic methods of assessing clinical response or progression are notoriously difficult in bone. CT and MRI scanning often do not show a change in tumor size despite subsequent histologic confirmation of necrosis after resection or obvious subjective clinical benefit. In some tumors, PET/CT helps to clarify relative metabolic activity but SUV quantification is also notoriously inaccurate in bone lesions. In our studies of osteosarcoma lesions, SUV by PET/ CT did not correlate either with absorbed dose to tumor or with time to progression [26]. Alternate imaging modalities, such as those that assess tumor blood flow or hypoxia, may ultimately prove to be of more benefit in assessing necrosis in bone lesions. Given the limitations of radiographic imaging in these lesions, it is our opinion that stable disease on radiographic studies should not be interpreted as a poor outcome and may represent potential benefit. Future trials should include overall survival and progression-free survival ought to include stable disease without relying on traditional RECIST criteria until a superior radiographic imaging modality is identified. Assessment and interpretation of serum biomarkers such as PSA, CA 15-3, or AP need further refinement. Particularly in patients with visceral or micrometastatic soft tissue disease apart from bone involvement, relying on a decrease in biomarkers as an index of response may underrepresent the local effects of ^153^Sm-EDTMP. Correlation with overall survival should be linked to any biomarker-based measure of progression. Finally, as addressed throughout this review, accurate dosimetry must be included with ^153^Sm-EDTMP treatment to confirm equivalent exposure to radiation prior to determining clinical response. Three-dimensional SPECT methods are ideal, as they also allow an assessment of the heterogeneity of uptake, a particular problem in larger tumors that may limit efficacy.

A second question is whether any patient or tumor characteristics predict benefit from ^153^Sm-EDTMP. Currently the drug is only approved for lesions that enhance on ^99m^Tc bone scan, however sporadic benefit has been described in patients with mixed osteoblastic/osteolytic lesions, especially in myeloma patients, and one woman with breast cancer experienced pain palliation despite lack of avid lesions on bone scan [54]. Two patients with osteosarcoma had extraosseous lesions that enhanced on bone scan and following ^153^Sm-EDTMP therapy these lesions developed significant (>95%) necrosis [26]. Therefore, other factors besides osteoblastic uptake may determine response to ^153^Sm-EDTMP. The importance of tumor histology remains poorly understood and further investigation of non-breast, non-prostate tumors treated with ^153^Sm-EDTMP may help to clarify this. Tumor microenvironment, particularly the tumor vascular network, is known to be critical for resistance to chemotherapy and radiation [38]. Hypoxia is well-described in many solid tumors including lung cancer and osteosarcomas and developing a means of identifying well-oxygenated tumors could help predict patients likely to have an effective response to radiation therapy. For example, soft tissue tumors of the lung and head and neck that appear to be hypoxic by [^18^F]-MISO (^18^fluoromisonidazole) PET scanning have been shown to respond poorly to radiation therapy [93,94]. If some metastatic bone tumors are more hypoxic than others, this could offer an explanation for the variability we have discussed in the clinical response to ^153^Sm-EDTMP treatment, including heterogeneous uptake of radiopharmaceutical, lack of consistent dose-response activity and dramatically different responses in patients despite similar histologies. Further understanding of the mechanism of pain palliation and skeletal uptake, particularly the cytokine environment, may help to optimize therapy and select patients who are likely to benefit. Finally, in patients with a rapid tempo of disease who also have soft tissue metastatic involvement, does treatment of the bone disease really help to delay progression, especially in combination with chemotherapy? The studies in prostate cancer seem to suggest that an improvement in progression-free survival may be possible, but further work is necessary.

Lastly, we have much to learn about how to optimally dose ^153^Sm-EDTMP and how to use adjunctive therapy in a synergistic manner. The data convincingly show that ^153^Sm-EDTMP is well-tolerated with predictable myelosuppression across a wide range of doses, including with repeated dosing and when followed by stem cell infusion. ^153^Sm-EDTMP appears to be synergistic in combination with chemotherapy, including docetaxel-based regimens in prostate cancer, bortezomib and melphalan in hematologic malignancies and gemcitabine in osteosarcoma. Combination with EBRT is also likely to become an effective strategy in prostate cancer and osteosarcoma. An unexplored realm is combining ^153^Sm-EDTMP with novel targeted therapies. Of particular interest is the combination with vascular-disrupting agents, especially in light of the questions raised regarding tumor hypoxia and impaired blood flow as a mechanism of radiation resistance and heterogeneity of radiopharmaceutical delivery [38]. It seems plausible that delivering ^153^Sm-EDTMP to a tumor with a crippled vascular infrastructure might help to optimize local cytotoxicity. Alternative strategies to consider also include immunotherapy, based on the upregulation of cancer-specific T-cells following exposure to ^153^Sm-EDTMP [69].

In summary, ^153^Sm-EDTMP is a well-tolerated radiopharmaceutical, with the potential to become a unique component of multimodality therapy for a wide variety of malignant bone tumors. Learning to use it effectively also promises progress in our understanding of tumor microenvironment and biology, as well as the mechanisms behind chemo- and radioresistance.

## Abbreviations

^153^Sm-EDTMP: ^153^Samarium Ethylene-Diamine-Tetramethylene-Phosphonic Acid
EBRT: External Beam Radio-therapy
IMRT: Intensity-Modulated Radiotherapy
Gy: Gray
mCi/ kg: Millicuries/Kilogram
MBq/kg: Megabecquerel/Kilogram
CRPC: Castrate-Resistant Prostate Cancer
SCT: Stem Cell Transplant
PSA: Prostate-Specific Antigen
CA 15-3: Cancer Antigen 15-3
AP: Alkaline Phosphatase
PAP: Prostate Acid Phosphatase
ADT: Androgen Deprivation Therapy
TBI: Total Body Irradiation
TBI: Total Body Irradiation
PFS: Progression-Free Survival
RAEB-T: Refractory Anemia With Excess Blasts In Transformation
AML: Acute Myeloid Leukemia
LGL: Large Granular Lymphocyte Leukemia
MTD: Maximally Tolerated Dose
